# High-Risk Prostate Cancer: A Very Challenging Disease in the Field of Uro-Oncology

**DOI:** 10.3390/diagnostics11030400

**Published:** 2021-02-26

**Authors:** Giorgio Napodano, Matteo Ferro, Roberto Sanseverino

**Affiliations:** 1 Department of Urology, Umberto I Hospital, ASL Salerno, 84014 Nocera Inferiore, Italy; giorgio.napodano@gmail.com (G.N.); roberto.sanseverino@libero.it (R.S.); 2 Department of Urology, European Institute of Oncology, IRCCS, 20141 Milan, Italy

Prostate cancer (PCa) is the most common cancer in males and affects 16% of men during their lifetime [1]. Up to 40% of patients are classified as high-risk (PSA ≥ 20 ng/mL or Gleason score 8–10 or clinical stage ≥T3) [2]. Despite improvement in patient selection and advances in treatment, disease recurrence remains substantial, affecting up to 50% of patients within 10 years and carrying a significant risk of progression and death [3,4]. Among non-curatively treated patients, 15 years mortality rate of a high-risk group is significant higher than intermediate and low risk groups (55% versus 20% versus 9%, respectively) [5]. Guidelines on the management of high-risk localized prostate cancer suggest two main options: radical prostatectomy (RP) with extended pelvic lymphadenectomy (ePLND), alone or in combination with adjuvant treatments, and radiotherapy (RT) associated with androgen deprivation therapy (ADT) [6,7,8]. Presently no prospective evidence exists comparing these two therapeutic options in this setting of patients.

Several studies have shown excellent oncological outcomes after RP: 10-year cancer-specific and overall survival ranges from 70% to 90% and from 58% to 83%, respectively. This wide range of prognosis depends on several factors, including biological heterogeneity, different definitions of high-risk group and number of removed nodes [9,10]. In these patients, an extended or superextended template allows the obtaining of an accurate staging and, probably, an improvement of oncological outcomes. In our experience, a retrospective analysis on 236 high-risk patients using a superextended lymphadenectomy including obturator, external iliac, common iliac, and hypogastric nodes showed improved staging compared to those with an extended lymphadenectomy (external iliac and obturator nodes), allowing the removal of a greater number of nodes [32.2 (± 9.1) versus 17.0 (± 8.6), *p* < 0.001], and the obtaining of more positive nodes (20.4% versus 2.3%, *p* < 0.001) without increasing risk of any complications (6.1% versus 22.5%, *p* < 0.05) and of lymphocele (0 versus 4.7%, 0.12). Furthermore, we must consider that approximately 40% of high-risk patients present a specimen-confined (≤pT3a, N0, R0) disease, hence they harbor a completely removable and more favorable disease and could mainly benefit from a surgical approach. Moreover, at least 30% of the patients do not need any adjuvant therapy [11]. Use of adjuvant radiotherapy (ART) and/or ADT is guided by evidence of positive surgical margins, extracapsular extension (pT3a/b), or lymph node metastases. Overall, three randomized controlled trials (SWOG 8794, EORTC 22911, ARO 9602) comparing adjuvant radiotherapy (ART) versus initial observation, confirmed that biochemical recurrence-free survival (BFS) was higher in patients with adjuvant radiotherapy than those without; however, only the SWOG trial demonstrated an advantage in terms of metastases-free survival (HR 0.71) though the number of events was very low (20 versus 37). Moreover, up to 40% of patients in the control arm did not experience any recurrence after 10 years; therefore, in this subset of patients, adjuvant treatment would represent an overtreatment in a not entirely negligible number of patients. These studies have also some relevant limits: radiation dose (60–64 Gy) is presently considered suboptimal, and in SWOG 8794 and EORTC 22,911 trials postoperative PSA was >0.2 ng/mL in 33% and 11% of patients, respectively; in these patients, radiotherapy truly represents a salvage rather than adjuvant treatment [12,13,14]. As mentioned, these three randomized trials have compared ART with observation and not with salvage radiotherapy (SRT) that should be currently considered to be valid alternative treatment. Although data from randomized trials are lacking, substantial evidence from retrospective studies shows that salvage RT is effective and reduces local recurrence and risk of distant metastasis and cancer-specific mortality [15]. Although a major determinant of the success of salvage radiotherapy is a low pretreatment PSA level, any specific PSA cut-off would be incorrect and inappropriate. Indeed, Fossati et al. reported that prognostic impact of PSA value varies according to pathological features (≥pT3b stage, Gleason score ≥8, positive surgical margins). In their study, the risk of biochemical recurrence in patients with ≤1 or ≥2 pathological risk factors increases by 1.5% and by 10% per 0.1 ng/mL of PSA, respectively [16]. Evaluating the role of hormonal therapy associated with postoperative RT, Fossati et al. found that only patients with 1 or ≥2 risk factors (≥pT3b stage, Gleason score ≥8, pre-RT PSA) benefit form a short (12-month) or long (36-month) course of ADT, respectively [17]. These findings seem to suggest that the use and timing of adjuvant therapy should be guided by the assessment of cancer aggressiveness analyzing a combination of multiple risk factors, to optimize the cost–benefit ratio of any postoperative treatments. However, to better define the role of adjuvant and salvage radiotherapy, results of 4 randomized controlled trials (Radicals, Raves, Getug 17, and EORTC 22043-30041) are expected soon.

Regarding primary radiotherapy treatment, European Association of Urology guidelines recommend external beam radiation therapy plus ADT with or without brachytherapy boost [6]. Some aspects of radiotherapy regarding dose, schedule, and duration of ADT still have not been defined. EBRT dose should be at least 76 Gy, because a correlation between oncological outcomes and dose of radiotherapy has been clearly demonstrated [18]. A hypofractioned schedule, consisting of fewer fractions than standard (about 19 versus 39), but with the same total dose, seems to improve patient convenience and logistic health care organization without compromising oncological outcomes [19]. Based on results of three randomized controlled trials (RTOG 9202, EORTC 22961, and DART 01/05), long (24–36-month) compared to short (4–6-month) ADT shows benefit in terms of overall survival [20,21,22]. However, a large meta-analysis of observational studies has suggested that ADT increases the risk of nonfatal cardiac events, suggesting a potential role in cardiovascular toxicity [23]. Moreover, in the control arm (short ADT) of RTOG 9202, about 70% of the patients were free of metastases after 20 years of follow-up. Therefore, we should wonder if all high-risk patients need a long-term ADT, or, rather, if it is more appropriate to identify those who could benefit from long-term ADT, despite risk of side effects and impairment to quality of life. Recently, Nabid et al. reported on 630 high-risk patients randomized to standard (36-month) or intermediate (18-month) ADT associated with EBRT (70 Gy on the prostate and 44 Gy on the pelvis); there were no differences in terms of overall survival, disease-specific survival, and biochemical failure between the two arms, but sexual quality of life, hot flushes, and testosterone recovery were significantly better in the intermediate ADT group. These findings suggest that 18 months of ADT could be an attractive and valid option, resulting in improved quality of life and reduced costs [24]. A viable option of radiotherapy treatment is represented by association of EBRT and ADT with brachytherapy (BT). Indeed, several retrospective studies have shown a benefit in terms of biochemical progression-free survival (BFS) and overall survival [25,26,27,28,29]. Results from three randomized controlled trials confirmed a significant advantage in BFS (HR 0.49; 95%CI 0.37–0.66), but not in overall survival, because in none of these studies was overall survival a primary endpoint [30,31,32]. These data should be prudently interpreted, because two trials [30,31] have relevant limits: EBRT was suboptimal due to only prostatic field and low dose (66 Gy [30], 55 Gy [31]), ADT was not performed in one study [30] and was too short (0 versus 6 versus 36 months) in the other one [31]. Regarding quality of life and tolerability, BT boost could be associated with higher risk of ≥G3 genitourinary and gastrointestinal toxicity, while interpretation of quality of life is complicated due to confounding factors such as ADT regimen and different radiation dose [33,34]. This evidence probably explains the low level of recommendation in the worldwide guidelines regarding use of BT in high-risk localized prostate cancer.

Therefore, in consideration of all this evidence, we advocate an urgent requirement for head-to-head comparison of surgery versus radiotherapy option, to improve our knowledge and to better counsel patients selecting personally preferred treatment. Results from randomized trials comparing RP versus EBRT + ADT are expected soon [35]. However, presently only retrospective and biased studies are available. Analyzing data on 42,765 patients with high-risk localized PCa diagnosed from 2004 till 2013 from the National Cancer Data Base, Ennis et al. found no significant difference in overall mortality between RP and EBRT + BT and ADT (HR 1.17 [95%CI, 0.88–1.55]). Patients that had undergone EBRT + ADT presented a higher risk of mortality than those treated with RP (HR 1.53 [95%CI, 1.22–1.92]) [36]. Successively, Berg et al. reanalyzed data from the aforementioned study, considering only young patients (<66 years) and with longer follow-up (92 months). Mortality risk was higher in 1702 EBRT + BT patients than in 12,283 RP patients (HR 1.22 [95%CI, 1.05–1.43]); however, absolute difference in overall mortality was only 3% [37]. These retrospective studies are invalidated by several limits, such as no information about adjuvant treatment, dose, and field of RT, duration of ADT, comorbidities and, especially, cause of death. Conversely, in another multicentric retrospective study on 1809 patients affected by high-grade (Gleason score 9–10) prostate cancer, EBRT + BT was associated with significantly lower 5-year prostate cancer-specific mortality compared with either radical prostatectomy or EBRT (3% versus 12% versus 13%, respectively; HR 0.38 [95%CI, 0.21–0.68] for RP and 0.41 [95%CI, 0.24–0.71] for EBRT) and 5-year distant metastases rate (8% versus 24% versus 24%, respectively; HR 0.27 [95%CI, 0.17–0.43] for RP and 0.30 [95%CI, 0.19–0.47] for EBRT). These results are weakened by relatively short follow-up (4.2–6.3 years), limited number (41%) of patients receiving adequate dose of EBRT and ADT, unknown impact of ADT on cancer mortality, and different follow-up management of participating centers [38]. Anyhow, it seems plausible that adding treatment could result in better outcomes, as claimed by Tilki et al., who found no difference in cancer-specific mortality between maxi RP (RP + ART + ADT) and maxi RT (EBRT + BT + ADT) (HR 1.33 [95%CI, 0.49–3.64]) [39]. However, it should be considered that adding treatment translates into higher complication rate, especially affecting sexual and urinary quality of life [40,41].

Although RP and RT are considered both equally valid options in the treatment of high-risk PCa, recently the surgery option has considerably increased, equaling radiotherapy rates [42,43]. In the absence of prospective and robust evidence, physicians should help high-risk patients to opt for their optimal and tailored treatment, considering both oncological outcomes and the safety profile of each option.
